# Draft genome sequence of endometrial *Alloscardovia omnicolens* strain Y28 identified in endometrial cancer

**DOI:** 10.1128/mra.01244-25

**Published:** 2026-02-10

**Authors:** Gwenllian A. Anwyl, Nicole R. Jimenez, Bonnie Hurwitz, Melissa M. Herbst-Kralovetz

**Affiliations:** 1 Department of Basic Medical Sciences, College of Medicine-Phoenix, University of Arizona 42283, Phoenix, Arizona, USA; 2 Department of Life Sciences, University of Bath 1555, Bath, United Kingdom; 3 Department of Obstetrics and Gynecology, College of Medicine-Phoenix, University of Arizona 42283, Phoenix, Arizona, USA; 4 Department of Biosystems Engineering, University of Arizona, Tucson, Arizona, USA; 5 University of Arizona Cancer Center 613590, Tucson, Arizona, USA; University of Pittsburgh School of Medicine, Pittsburgh, Pennsylvania, USA

**Keywords:** *Alloscardovia omnicolens*, endometrial cancer, endometrial microbiota, human microbiome, gynecologic health

## Abstract

We report a 1.8 Mb draft genome of *Alloscardovia omnicolens* Y28 isolated from a patient with endometrial cancer.

## ANNOUNCEMENT

*Alloscardovia omnicolens* was first identified in Europe in 2007, following isolation from diverse human samples, including the urogenital tract (1), bloodstream (2), oral cavity, and abscesses of the lung and aortic valve (2). It is a gram-positive, non-spore-forming, non-motile rod that grows facultatively anaerobically, occurring singly, as pairs, or chains (2). In clinical samples, *A. omnicolens* is frequently co-isolated with *Bifidobacterium spp.* (3). Although usually considered commensal (4*), A. omnicolens* has been associated with urinary tract infections and bacteremia (1), suggesting an opportunistic role.

This announcement documents the draft genome of the endometrial *A. omnicolens* strain Y28, clarifying its genomic characteristics and evolutionary relationship, to advance understanding of endometrial microbiota.

As part of a larger multi-omic study on endometrial cancer and benign condition (5–7), this research project was conducted at the University of Arizona. The Institutional Review Board approved the study (reference no. 1708726047), and the participant diagnosed with endometrial cancer provided written informed consent. Endometrial samples were collected from the bifurcated uterus using sterile swabs. Swabs were stored in Amies transport media with 10% glycerol, after which microbial isolates were obtained and cultured under anaerobic conditions at 37°C on Tryptic Soy Agar supplemented with 5% sheep’s blood for 48 h. Bacterial DNA was extracted using the Qiagen DNeasy PowerSoil Pro kit (MO BIO Laboratories, Carlsbad) and sequenced at the University of Arizona PANDA Core for Genomics & Microbiome Research. Paired-end sequencing was performed using Illumina’s PCR-Free Library Prep and the NextSeq 1000 Platform (300-cycle) with a 2×150 bp read length. Trimmomatic (v0.39) (8) improved read quality and was assessed with FastQC (v0.11.9) (9). Kraken2 (v2.1.3) (10) and Bracken (v2.8) (10) were used for species-level read classification based on the k2_pluspf database (downloaded on 2023-06-05) (11). Krakentools (v1.2) (10) (extract_kraken_reads.py, --taxid 9606, --include-children) was used to separate human from microbial reads. Assembly utilized Unicycler (v16.0) (12) using SPAdes (13), followed by quality checks with Checkm2 (v1.0.1, -m 500) (14) and Quast (v5.2.0) (15). Annotation was performed using PGAP (v 6.1) (16). Default parameters were used for all tools unless otherwise specified. All code is available on GitHub (https://github.com/hurwitzlab/vaginal_genome_assembly). Genomic analyzes were performed using the Bacterial and Viral Bioinformatics Resources Center (17, 18).

The draft of *A. omnicolens* Y28 comprises 13 contigs, with 1,844,906 bp, an N50 of 1,026,604, and a GC content of 46.84% (Table 1). Genome annotation revealed 1,568 coding sequences (CDS), including 1,536 functionally assigned proteins and 32 hypothetical proteins, alongside 46 tRNA and 5 rRNA genes (Table 1).

**TABLE 1:** Genomic assembly and characteristics of *A. omnicolens* strain Y28^*a*^

Genome name	*A. omnicolens* Y28
Isolate Information	
Isolation Source	Endometrium
Health Status	Endometrium Cancer
Strain Identity	
BV-BRC Genome Similarity	*A. omnicolens* DSM 21503
Genome Similarity Assembly Accession	GCA_002259705.1
K-mer Count	4/1000
K-mer Distance	0.23011
Average nucleotide identity to the Representative strain (%)	98.80
Genome characteristics	
Genome size (bp)	1,844,906
Number of Contigs	13
Total Raw reads	1,195,459
Contig N50 (bp)	1,026,604
Contig L50	1
GC (%)	46.84
Genome coverage	200×
Number of 5S rRNA	1
Number of 16S rRNA	2
Number of 23S rRNA	2
Number of tRNA	46
Number of CDS	1,568
Number of CDS with functional assignments	1,536
Number of Unique protein families	8
Unique protein Families IDs (PGFAMs-Hypothetical)	PGF_05514654; PGF_04425848; PGF_01749992; PGF_05057205; PGF_05115053; PGF_10607971; PGF_01750103; PGF_05158714
Unique protein Families IDs (PGFAMs- Non-Hypothetical)	0
Number of Antibiotic Resistance Gene IDs (PATRIC)	20
Antibiotic Resistance Gene IDs	MtrA; gyrB; gyrA; S10p; Alr; Ddl; MurA; gidB; rpoB; MtrB; S12p; rho; Ddl; Iso-tRNA; foIP; EF-G; EF-Tu; PgsA; foIA, Dfr; rpoC

^*a*^ The genome of *A. omnicolens* was isolated from the endometrium of a patient with endometrial cancer. Genus and species identification was performed by using genome similarity and validated by Average Nucleotide Identity. The table summarizes genome assembly statistics, including the number of contigs, total raw reads, N50, genome size, and GC content, as well as genome annotation results. Key genomic features reported include the number of coding sequences (CDS), rRNA and tRNA genes, as well as the proportion of genes with functional assignments. Comparative analysis with reference strain *A. omnicolens* DSM 21503 is provided, along with average nucleotide identity (ANI) and k-mer metrics. Additionally, utilizing BV-BRC we identified the number of protein families and unique protein families identified by PATRIC, and predicted antibiotic resistance genes and their corresponding PATRIC gene identifiers.

**Fig 1:**
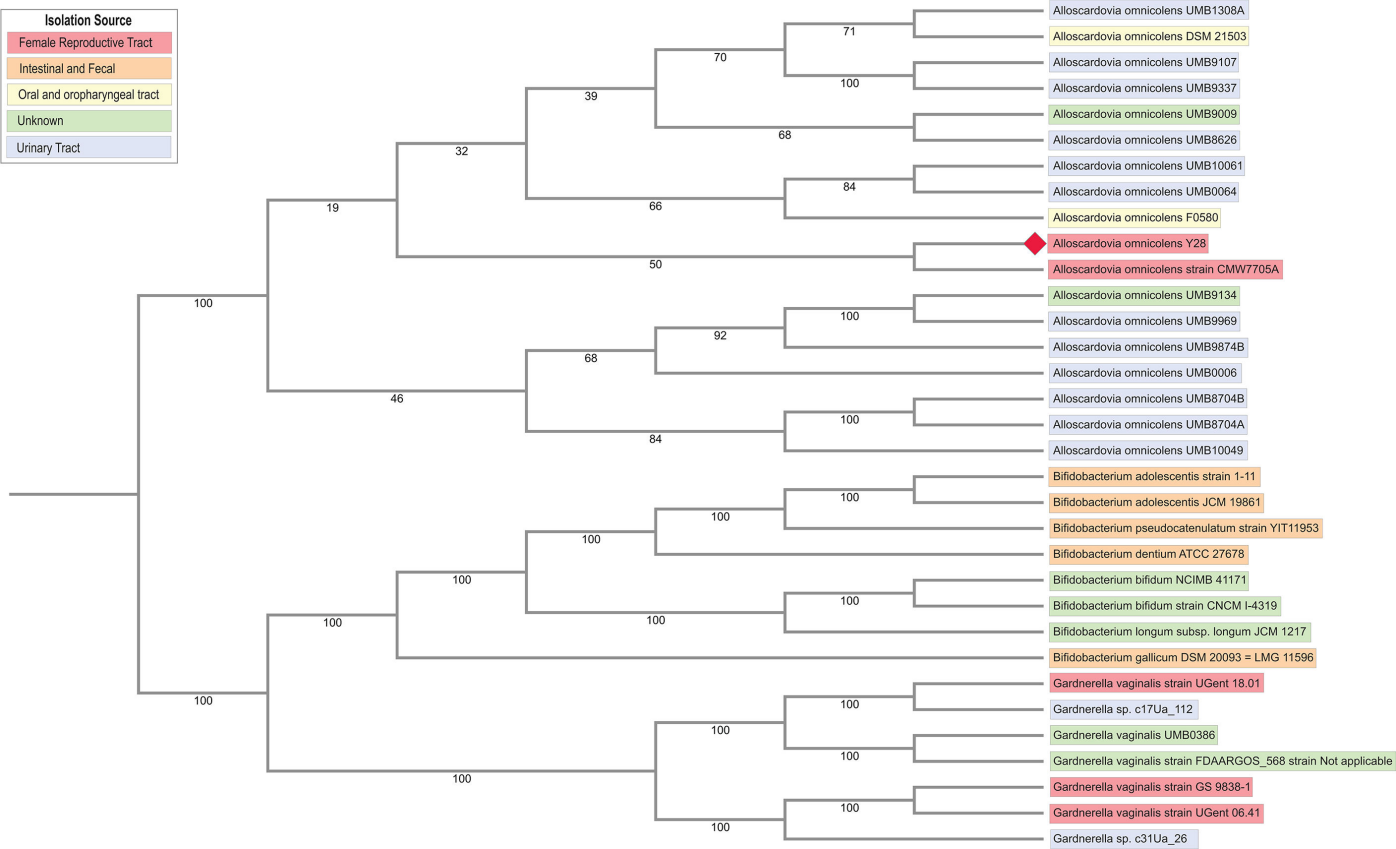
Single Copy Orthologous Phylogenetic Tree amongst publicly available *A. omnicolens* strains, Y28 and related *Bifidobacteriaceae* Species. Single-copy orthologous genes and RaxML were used to create the phylogenetic tree (*n* = 415) and were aligned by codon using MUSCLE. The tree includes 34 whole genomes, all from human hosts. *A. omnicolens* strain Y28 is discussed in this microbial resource announcement and indicated by a red diamond. Colors of strain names are based on their isolation source. Bootstrap values (shown at nodes) reflect the robustness of each branching point.

Strain Y28 belongs to the *Alloscardovia* genus, sharing 98.8% nucleotide identity with the representative strain (Table 1) also observed in the phylgenetic tree (Fig. 1). RASTtk (19) identified that Y28 shared 1,048 protein families with publicly available *A. omnicolens* whole-genome strains but also possessed eight unique hypothetical proteins (Table 1). Amino acid metabolism represented 35.5% of the metabolism-associated genes, with high levels of lysine, threonine, and cysteine pathways (28 genes). Such metabolic alterations are increasingly recognized in gynecologic health (20, 21), where disruptions in these pathways may contribute to endometrial cancer (22). The presence of these pathways may reflect adaptations to the endometrial niche, suggesting potential interactions with the host.

## Data Availability

The draft genome sequence of A. omnicolens Y28 has been deposited at the Sequence Read Archive (SRA) under accession number NZ_JBRAVA000000000. This genome is part of a larger BioProject PRJNA1354867. The genome BioSample identification is SAMN51299389. The genome annotation can also be found at GenBank GCF_052772085.1, and additional genome assembly, annotation, protein family, and phylogenetic analysis information are available at BV-BRC (https://www.bv-brc.org/workspace/jimeneznr@patricbrc.org/Alloscardovia) with access via a free account. Additional assembly information and parameters utilized can be found at https://github.com/hurwitzlab/vaginal_genome_assembly.
